# Global Future Modeling of the Monophagous Pest *Pagiophloeus tsushimanus* (Coleoptera: Curculionidae): An Integrated Approach Using Random Forest and the CLIMEX Model

**DOI:** 10.3390/insects17101008

**Published:** 2026-09-30

**Authors:** Qiang Wu, Kaitong Xiao, Xiaosu Deng, Haijian Hu, Juan Li, Zhenxi Lin, Hang Ning

**Affiliations:** 1 Hubei Key Laboratory of Biological Resources Protection and Utilization, Hubei Minzu University, Enshi 445000, China; lxwq20030226@163.com (Q.W.); 13402709191@163.com (K.X.); 18785237443@163.com (X.D.); 18672074599@163.com (H.H.); ljzinnia2019@163.com (J.L.); l13432467103@163.com (Z.L.); 2 College of Forestry and Horticulture, Hubei Minzu University, Enshi 445000, China

**Keywords:** *Cinnamomum camphora*, host plant constraint, invasive weevil, shared socioeconomic pathways, biosecurity, phytosanitary risk

## Abstract

*Pagiophloeus tsushimanus* Morimoto, 1982, a camphor tree weevil, is an emerging invasive wood-boring pest that has resulted in a progressive decline of *Cinnamomum camphora* plantations in affected areas. Native to Tsushima Island, Japan, *P. tsushimanus* has also spread to eastern China. With global climate change, the geographic range, occurrence frequency, and severity of this pest are continuously increasing, particularly in affected areas of China. Therefore, to limit the continued spread of this pest, the Random Forest (RF) algorithm and the CLIMEX model were combined to map the potential global distribution of *P. tsushimanus*. The results indicated that the potential range of *C. camphora* was jointly regulated by moisture availability (BIO18) and human modification indicators (PD and gHM). In contrast, the geographic distribution limits of *P. tsushimanus* were primarily defined by cold stress thresholds at higher latitudes and by heat and dry stress limitations in its traditional subtropical core range. Under current climatic conditions, the host-constrained suitable habitat of *P. tsushimanus* covered 4238.03 × 10^4^ km^2^, mainly in East Asia, the southeastern United States, southeastern South America, and the Mediterranean coast of Europe. Under future climate change scenarios, *P. tsushimanus* and its host, *C. camphora*, exhibit a highly convergent poleward range expansion. New suitability hotspots for *P. tsushimanus* were projected to emerge in central and northern China, western and central Europe, and the northeastern United States. Owing to increased heat and drought stress in low-latitude subtropical core areas, the global suitable area under future climate change contracted by 17.99% overall, and highly suitable habitat decreased by 39.22%. The suitability maps of *P. tsushimanus* generated under host-constrained conditions in this study provide a potential early-warning layer for global forest health. We recommend that forestry departments in newly suitable temperate zones implement strict phytosanitary inspections of imported *C. camphora* nursery stock and that the timber and forest-product industries establish active monitoring programs to prevent the accidental introduction and establishment of this cryptic wood-boring pest.

## Simple Summary

*Pagiophloeus tsushimanus* is a highly destructive wood-boring pest that threatens camphor trees (*Cinnamomum camphora*), ecologically and economically important landscaping trees in East Asia. To project its potential global spread, we mapped its potential distribution by integrating the ranges of both the pest and its host via Random Forest and CLIMEX models. We found the host’s distribution is primarily constrained by moisture-related factors (precipitation deficit and warm-season precipitation) and human modification. Under current climate, the total host-constrained suitable habitat for the pest reaches 4238.03 × 10^4^ km^2^, mainly concentrated in East Asia, the southeastern US, southeastern South America, and the Mediterranean coast of Europe. Under future climate change, both species will shift northward, forming new high-risk invasion zones in Northern China, Western Europe, and the northeastern US. Meanwhile, rising heat and drought stress will reduce suitability in traditional subtropical ranges, leading to an overall 17.99% contraction in global suitable area. These findings provide a realistic risk map to help forest managers and customs target quarantine inspections and prevent the global spread of this weevil.

## 1. Introduction

*Pagiophloeus tsushimanus* Morimoto, 1982 (Coleoptera: Curculionidae), the camphor tree weevil, is a recently recorded wood-boring pest in China and was first reported in 2014 [1,2]. This beetle was first discovered on Tsushima Island, Japan, in 1982 [3]. Owing to the lack of substantial adverse effects in the local area and the absence of evidence of spread beyond the island, it has attracted limited academic interest domestically and internationally [2]. In 2006, during an investigation of garden pests and diseases in Shanghai, *P. tsushimanus* was first discovered in China, and its detrimental effects have subsequently intensified [1,2]. In 2014, this pest was officially identified as a newly recorded species in China [1]. Currently, camphor trees in various administrative districts of Shanghai are severely damaged by this pest, and its distribution continues to expand [2,4]. Because the larvae of this pest remain concealed within the trunks of *Cinnamomum camphora* (L.) J. Presl (Lauraceae), the pest has a cryptic life cycle, causes substantial damage, and is difficult to control, seriously affecting the normal growth of *C. camphora* and potentially resulting in tree mortality [2,5]. In wild conditions, *P. tsushimanus* feeds only on camphor trees and completes its life cycle on this host, making it a highly specialized herbivorous insect [5,6]. Specifically, it is a strictly monovoltine species, with both larvae and adults overwintering within the branch forks or wood tunnels of the host plant [2]. Field investigations have demonstrated that *P. tsushimanus* is mainly distributed in pure plantations of *C. camphora*, which provide favorable conditions for the establishment and rapid expansion of the pest population.

*Cinnamomum camphora*, a camphor tree, is a key evergreen timber and ornamental species in East Asia with substantial ecological, economic, and cultural significance [7]. Characterized by its broad environmental adaptability and high tolerance of urban anthropogenic stress, *C. camphora* is extensively used in urban afforestation, landscape management, and ecological restoration programs [7]. However, the rapid expansion of monoculture camphor plantations and interconnected urban green corridors has provided extensive resource pathways for specialized herbivores, thereby increasing the potential for pest outbreaks and regional range expansion [8]. Presently, *P. tsushimanus* has established stable populations in all 14 administrative regions of Shanghai [2]. Additionally, with global climate change, the geographic range, occurrence frequency, and severity of this pest are continuously increasing, particularly in affected areas of China [9]. Although adults disperse naturally by flight, its cryptic wood-boring lifestyle facilitates passive, long-distance interprovincial and international dissemination [10]. Eggs, larvae, and pupae concealed within infested timber or landscape nursery stock can be readily transported through global trade and horticultural supply chains [10,11]. Therefore, owing to the increasing biosecurity challenges associated with this pest, elucidating the potential global distribution and environmental thresholds of *P. tsushimanus* at the global scale is necessary to provide an important basis for the development of proactive warning and quarantine protocols for this pest.

Global warming, as documented in the Intergovernmental Panel on Climate Change (IPCC) Sixth Assessment Report, exerts profound thermal constraints on the distribution and population dynamics of ectothermic insect pests [12,13]. Temperature directly regulates the metabolic rate, reproductive output, and developmental rate of insects, altering their population dynamics and geographical distributions [9,13]. Climate change influences the geographical range of forest insects through three primary ecological pathways. First, increasing temperatures reduce low-temperature lethal thresholds in high-latitude and high-altitude regions, facilitating poleward and upward range expansion into areas previously protected from cold stress [14]. Second, accelerated thermal accumulation can shorten life cycles and increase annual generation number, potentially intensifying local pest pressure [9,14]. Third, extreme summer heatwaves and altered precipitation regimes may exceed the physiological tolerance of insects, leading to thermal stress, habitat degradation, and range contraction in lower-latitude tropical zones. Field observations have recently revealed that the geographic range and occurrence frequency of *P. tsushimanus* have been continuously increasing with higher temperature [2]. Host plant availability and human activity are key factors in determining the geographical distribution of herbivorous pests [15]. As a monophagous insect, the effects of these factors on the geographical distribution of *P. tsushimanus* are self-evident. Changes in the host distribution may fundamentally affect the distribution of this pest. Anthropogenic factors such as rapid urbanization and intensive domestic transportation of live nursery stock can bypass natural dispersal barriers and accelerate the establishment of pests in human-dominated landscapes [10,16]. In summary, these factors may all contribute substantially to changes in the geographical distribution of *P. tsushimanus*. Therefore, understanding how the geographical distribution of *P. tsushimanus* responds to these factors is critical for enabling appropriate ongoing forest ecosystem management.

Species distribution models (SDMs) have emerged as important tools for forecasting invasion risk and supporting quarantine programs [17]. SDMs are classified into two categories: correlative and mechanistic [18]. Correlative models, such as MaxEnt and Random Forest (RF), leverage the statistical relationship between species occurrence and environmental data, assuming that the current distribution accurately indicates the species’ ecological requirements [19]. Currently, MaxEnt and RF models, which have simple construction, low computational cost, and strong predictive performance within the environmental parameters defined by the training data and are based on machine learning algorithms, are widely used to project the potential distribution range of invasive species [20]. RF is used to capture complex, nonlinear relationships and high-dimensional interactions between species occurrence and diverse environmental or anthropogenic variables [20]. CLIMEX, a typical mechanistic model that does not depend on equilibrium assumptions, uses physiological parameters and climatic tolerance to define the fundamental niche of a species, with historical occurrence data used for parameterization [21]. However, mechanistic models require numerous parameters and substantial amounts of physiological and ecological data that are difficult to acquire [22]. Many studies have reported limitations in predictions based on a single SDM. Therefore, to enhance the accuracy and credibility of model predictions, an increasing number of studies have combined correlative and mechanistic models [23,24]. Consequently, RF and CLIMEX were selected to model the future distribution of *P. tsushimanus*.

In this study, we integrated the RF and CLIMEX models to evaluate the potential global distribution of *P. tsushimanus* under current and future climate scenarios. RF was used to project the potential distribution of the host, *C. camphora*, while CLIMEX delineated the climatic suitability limits of the pest based on the distribution constraints of the host. Specifically, this study aimed to (1) explore the potential global distribution of *P. tsushimanus* and *C. camphora* under the current climate scenario and determine the key factors affecting their distribution, (2) predict their potential global distribution under future climate scenarios, (3) estimate possible expansion or contraction of the species’ range with global warming, and (4) obtain the necessary theoretical basis for monitoring, early warning, and effective prevention and control of the pest, thereby providing an important reference for controlling the spread of *P. tsushimanus*.

## 2. Materials and Methods

### 2.1. Overall Modeling Workflow

First, RF was used to project the potential distribution of *C. camphora*. For the host *C. camphora*, which was extensively planted, there were high-density occurrence records. The distribution range of *C. camphora* is influenced by human planting preferences, urbanization, and transportation networks [7,8]. Therefore, RF was selected to capture the complex, high-dimensional, and nonlinear relationships between species occurrence and multidimensional predictors, and to effectively integrate environmental factors and human activity variables [20]. Second, CLIMEX 4.0.2 was used to project the potential global distribution of *P. tsushimanus* [25]. For this pest, field occurrence records are currently limited, which can introduce substantial spatial sampling biases into the modeling process [26]. Additionally, the developmental thresholds and physiological parameters of *P. tsushimanus* have been well characterized in laboratory studies [2,27]. Third, owing to the obligate dependence of *P. tsushimanus* on its host, we used the global host potential distribution as a mask to extract the global potential distribution of *P. tsushimanus*, representing the host-constrained potential global distribution of *P. tsushimanus* [28].

### 2.2. Acquisition and Curation of Species Occurrence Records

The occurrence records of *P. tsushimanus* were compiled from three primary sources: public biodiversity databases, the literature, and field investigations. Three georeferenced occurrence records were obtained from the Global Biodiversity Information Facility (GBIF Occurrence Download. Available online: https://doi.org/10.15468/dl.4sxpgp (accessed on 2 June 2026)) [29]. This dataset was supplemented with five historical occurrence records extracted from the relevant literature [1,2,3]. To obtain more occurrence records for *P. tsushimanus*, field investigations were conducted from 2024 to 2025 in collaboration with the Forestry Protection Department. Locality names, precise coordinates, and elevations were recorded for each occurrence site. Duplicate and spatially redundant records were removed to reduce potential prediction errors associated with sampling bias. For historical records lacking precise coordinates, manual georeferencing was performed using Google Maps (version 25.38.02), based on descriptive locality information, to assign latitude and longitude coordinates [30]. A final dataset comprising 17 highly reliable occurrence (Table S1) records for *P. tsushimanus* was obtained.

**Figure 1:**
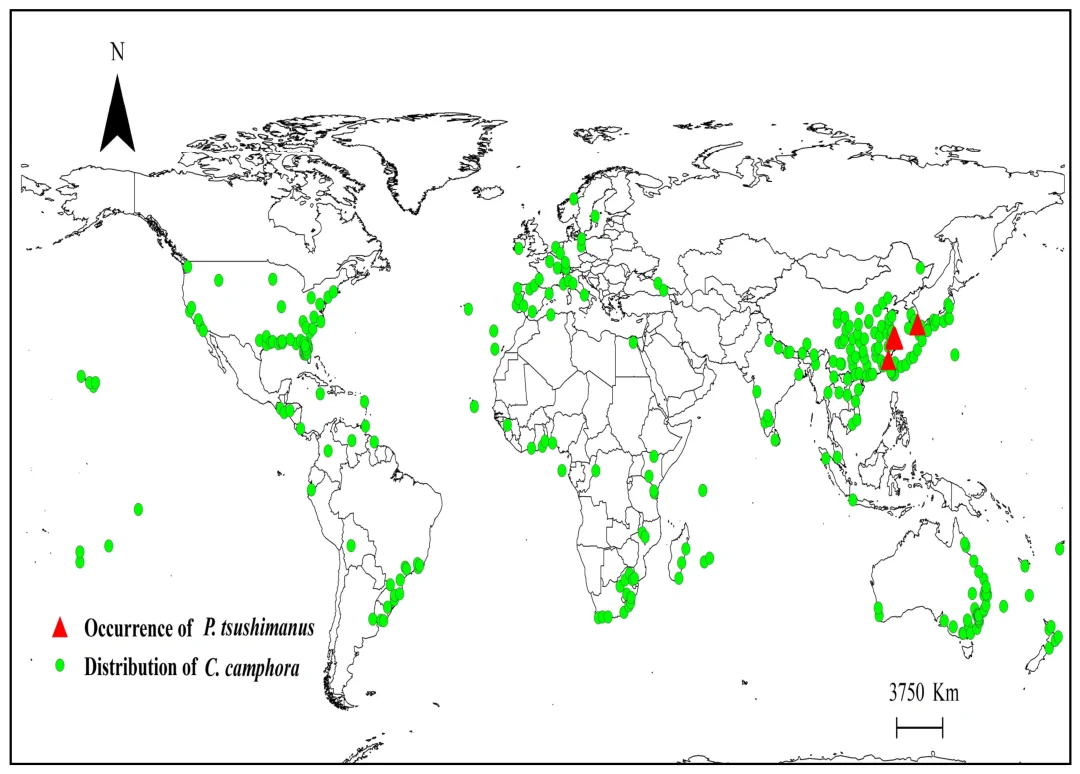
Global presence coordinates of *P. tsushimanus* and *C. camphora*.

The occurrence data for *C. camphora* were compiled and curated using a parallel methodology. First, a raw dataset comprising 17,395 occurrence records was compiled [31,32,33]. Owing to intensive cultivation and localized documentation efforts, these raw records exhibited high spatial clustering, which could introduce substantial spatial autocorrelation and sample selection bias [26,34]. To mitigate these biases and reduce spatial dependence, the raw dataset was spatially filtered using the “Spatially Rarefy Occurrence Data” tool in the SDM Toolbox for ArcGIS 10.8 with a 10-arcminute (~18.6 km) search radius [35]. Following this thinning process, a final dataset of 1888 spatially rare field host plant occurrence (Table S2) records was retained for subsequent spatial modeling. To prevent visual overcrowding and ensure cartographic clarity on the global-scale map, these 1888 modeling points were further thinned to 300 representative coordinates (Table S3) solely for geographic visualization (Figure 1).

### 2.3. Using RF to Project the Potential Global Distribution of C. camphora

#### 2.3.1. Bioclimatic and Anthropogenic Data

Nineteen bioclimatic variables were obtained from the WorldClim database (https://www.worldclim.org/ (accessed on 26 May 2025)) at a 10-arcminute spatial resolution [36]. Three anthropogenic variables, namely Global Human Modification (gHM), Human Footprint (HF), and population density (pop), were incorporated as predictors. The gHM layer was acquired from the Figshare open repository (https://figshare.com/ (accessed on 22 May 2025)), the HF layer was sourced from the Stanford Natural Capital Project (http://naturalcapitalalliance.stanford.edu/ (accessed on 22 May 2025)), and population density was obtained from the WorldPop database (https://hub.worldpop.org/ (accessed on 22 May 2025)) [37,38,39]. The 22 variables are shown in Table 1. All three anthropogenic layers, originally generated at a 30-arcsecond (~1 km) resolution, were resampled to a 10-arcminute spatial resolution using bilinear interpolation to ensure spatial alignment with the bioclimatic predictors.

For future projections, the IPCC Sixth Assessment Report (AR6) framework, which uses the Shared Socioeconomic Pathways (SSPs), was applied [40]. To maintain methodological consistency with the medium-level emission scenario (A1B) used in the CLIMEX pest modeling, the SSP3-7.0 scenario (representing a medium-to-high greenhouse gas emission pathway under the regional rivalry trajectory) was selected for the 2050s host plant simulations. The 2050 population density dataset under the SSP3 scenario was used to correspond to the SSP3-7.0 climate scenario [41].

#### 2.3.2. Modeling Process

To simulate the potentially suitable distribution of the host plant *C. camphora*, RF was implemented via the biomod2 ensemble platform in the R (version 4.4.1) statistical computing environment (Script S1) [42,43]. Pseudo-absence points were generated to mitigate class imbalances and to define the environmental background for model training. These points were randomly sampled across the entire study area, with a 10-km buffer zone surrounding all verified occurrence records excluded from sampling to account for spatial sampling uncertainty and positional errors. Following a 1:4 presence-to-absence ratio relative to 1888 filtered host occurrence records, 7552 pseudo-absence points were generated to construct a presence–pseudo–absence dataset for model calibration [44]. The 1:4 presence-to-absence ratio was adopted because the RF machine-learning algorithm exhibits exhibit optimal predictive accuracy and reduced classification bias when calibrated with a larger background sample size [44]. The 10-km exclusion buffer around presence records was implemented to minimize commission errors by preventing pseudo-absences from being selected in areas where the host is highly likely to occur but remains undocumented. A global modeling extent was utilized to fully capture the macroclimatic niche and realized environmental limits of *C. camphora*, which has been widely naturalized outside its native East Asian range. During model training, the optimal hyperparameters of the RF model (specifically the number of variables randomly split at each node, mtry) were optimized using a random search with 5-fold cross-validation implemented via the caret package in R.

A 10-fold cross-validation strategy was used to evaluate the predictive performance of the model. In each fold, the host occurrence dataset was randomly split; 70% of the records were used for model training, and the remaining 30% were retained as an independent test set. This cross-validation procedure was repeated ten times to reduce stochastic fluctuations and improve predictive stability. Furthermore, the relative contributions of each bioclimatic and anthropogenic variable in shaping the ecological niche of *C. camphora* were quantified using the intrinsic feature importance scores of RF, providing a quantitative basis for identifying the key environmental predictors of host plant distribution.

#### 2.3.3. Evaluation of Model Accuracy

The RF performance was assessed using three widely accepted metrics: the Area Under the Curve (AUC) of the Receiver Operating Characteristic (ROC), the True Skill Statistic (TSS), and the kappa coefficient [45]. The AUC ranges from 0 to 1, with values exceeding 0.90 indicating excellent discriminatory capacity [46]. The kappa coefficient evaluates agreement between observed and predicted classifications based on the confusion matrix, ranging from −1 to 1 [45]. As a robust alternative to the kappa statistic, the TSS evaluates the model’s discrimination capacity by calculating the difference between sensitivity and specificity, with scores ranging from 0 to 1 [47]. For all three indices, values closer to 1 indicate higher predictive reliability.

#### 2.3.4. Classification of Suitability

To characterize host habitat suitability more intuitively, the continuous probability outputs (ranging from 0 to 1) generated by RF were classified into five suitability tiers: unsuitable, very low, low, medium, and high suitability. Initially, the Natural Breaks (Jenks) algorithm in ArcGIS 10.8 was applied to calculate the preliminary classification thresholds [48]. Because Jenks’ method operates solely on statistical variance (maximizing between-class variance while minimizing within-class variance), these mathematically determined thresholds were further adjusted and empirically calibrated by overlaying the verified occurrence records of *C. camphora*. This refinement process was intended to align the final suitability classifications with the host’s observed geographic distribution, thereby reducing potential classification errors (ensuring that most of the verified occurrence records were within suitable categories, whereas the unsuitable class represented areas with low predicted suitability) [49].

### 2.4. Using CLIMEX to Project the Potential Global Distribution of P. tsushimanus

#### 2.4.1. CLIMEX Model

CLIMEX was used to project the potential global distribution of *P. tsushimanus*. In this framework, the ecoclimatic index (EI) was used as a measure of climatic suitability for the species at a given location. The EI was calculated based on the annual growth index (${\mathrm{GI}}_{A}$), stress indices (SI), and interactive stress (SX), along with two limiting factors: the accumulated degree-days required to complete a life cycle (PDD) and the diapause index (DI). ${\mathrm{GI}}_{A}$ indicates the potential for population growth under suitable growth conditions and is primarily affected by the temperature index (TI) and moisture index (MI). ${\mathrm{GI}}_{A}$ is a dimensionless, unitless index scaled from 0 to 100 that represents relative climatic suitability for population growth rather than any physical measurement of biomass, trunk surface area, or tree count. SI, which includes cold stress (CS), heat stress (HS), dry stress (DS), and wet stress (WS), indicates the degree of population decline during unfavorable seasons. SX was not included in the calculations. The EI value ranges from 0 to 100, indicating climatic suitability for the species, and can attain a maximum of 100 under optimal climatic conditions.

$${\mathrm{EI}=\mathrm{GI}}_{A}\;\times \;\mathrm{SI}\;\times \;\mathrm{SX}$$

SX, the Stress Interaction Index, is typically disregarded. ${\mathrm{GI}}_{A}$ is primarily affected by the Temperature Index (TI) and the Moisture Index (MI). The calculation formula is as follows:

$${\mathrm{GI}}_{A}\;=\;\mathrm{TI}\;\times \;\mathrm{MI}$$

SI includes cold stress (CS), heat stress (HS), dry stress (DS), and wet stress (WS), expressed as follows:

$$\mathrm{SI}\;=\;(1-\frac{\mathrm{CS}}{100})\;\times \;(1-\frac{\mathrm{HS}}{100})\;\times \;(1-\frac{\mathrm{DS}}{100})\;\times \;(1-\frac{\mathrm{WS}}{100})$$

#### 2.4.2. Meteorological Data

The CLIMEX model requires meteorological inputs, structured as location (.loc) and meteorology (.met) files, which were compiled into MetManager (.mm) databases [25]. Standard meteorological parameters used in the .met files included daily minimum temperature (Tmin), daily maximum temperature (Tmax), monthly total precipitation, and relative humidity recorded at 09:00 (RH0900) and 15:00 (RH1500).

To characterize the climatic suitability for *P. tsushimanus* under historical and future scenarios, gridded climate datasets at a 10-arcminute spatial resolution were retrieved from the CliMond database (http://www.climond.org/ (accessed on 22 May 2024)) [50]. Standard-formatted climate files compatible with CLIMEX were acquired directly from the platform. For the historical baseline period (1961–1990, centered on 1975), data were derived from WorldClim and Climate Research Unit (CRU) climatological normals (CL1.0, CL2.0) [36,51].

For future climate projections, because standardized future datasets natively formatted for CLIMEX are structurally restricted to the emission scenarios of the IPCC Fourth Assessment Report (AR4), the SRES A1B emission scenario (representing a medium-level greenhouse-gas emission pathway) was selected for the 2050s projection of *P. tsushimanus* [52]. The 2050s, representing the 2041–2060 climatological normal, was selected as the target future period because it serves as a critical mid-term planning window for forest biosecurity and urban afforestation management. Furthermore, climate projections for the mid-century exhibited lower model divergence and cumulative uncertainty among greenhouse gas pathways than those for far-future horizons (the 2080s or 2100), thereby yielding more robust and actionable predictions for long-lived perennial host species such as *C. camphora* [53].

#### 2.4.3. Parameter Calibration and Validation

To predict the potential distribution of *P. tsushimanus*, the CLIMEX model was parameterized and validated as follows: preliminary parameter values were derived by adapting a representative climatic template to the native habitat of the species (East Asia, including Japan) and incorporating available biological data. Parameter calibration was performed through an iterative fitting procedure to minimize discrepancies between the predicted climate suitability and observed distribution patterns [25]. Given the extremely limited global distribution of this newly emerging pest, model evaluation was conducted by assessing the predictive sensitivity across the entire dataset of 17 occurrence records, as partitioning such a small sample size would yield an insufficient testing set. The biological and empirical rationale behind each fitted parameter is provided below, and the final parameter configurations are summarized in Table 2. To evaluate the robustness of our CLIMEX projections, we conducted a parameter sensitivity analysis on the core parameters, including the lower temperature threshold (DV0), lower soil moisture threshold (SM0), and cold stress temperature threshold (TTCS). Each parameter was individually adjusted by ±10% (specifically, ±1 °C for temperature thresholds and ±0.05 for moisture thresholds) while keeping all other parameters constant. The results demonstrated that the model was highly stable and robust. Minor variations in these core parameters led to minimal fluctuations (<5%) in the total projected suitable area, and all 17 verified contemporary distribution records of *P. tsushimanus* consistently maintained suitable Ecoclimatic Index (EI) values (EI > 10).

Temperature index (TI): The TI was defined by four critical temperature thresholds: the lower temperature threshold (DV0), lower optimum temperature (DV1), upper optimum temperature (DV2), and upper temperature threshold (DV3). These parameters were established by integrating the experimental observations of *P. tsushimanus* with its known geographical distribution. According to laboratory rearing studies by Jia et al., the lower developmental threshold temperatures for the egg, larval, and pupal stages of *P. tsushimanus* were 9.03, 13.04, and 10.32 °C, respectively [27]. As the larval stage exhibits the highest developmental threshold, which acts as a physiological limitation to complete life-cycle development, DV0 was set at 13.04 °C. The optimum developmental temperature range for this species is documented to occur between 20 and 28 °C; consequently, DV1 and DV2 were set at 20 and 28 °C, respectively [27]. DV3 was set at 33 °C, indicating the temperature at which survival and developmental rates begin to decline under heat stress.

Moisture index (MI): The MI consisted of four soil moisture thresholds: the lower threshold (SM0), lower optimal moisture (SM1), upper optimal moisture (SM2), and upper threshold (SM3). As a monophagous pest, the survival of *P. tsushimanus* is highly dependent on its host, *C. camphora*, which thrives in warm, humid, subtropical climates with annual precipitation exceeding 1000 mm [7,33]. Considering that camphor trees prefer moist, well-drained soils and cannot tolerate prolonged drought, SM1 and SM2 were set to 0.6 and 0.8, respectively, representing optimal soil moisture levels for host forest growth. SM0 was set at 0.1 to define the lower limit of soil moisture below which the insect cannot survive owing to host desiccation, while SM3 was set at 1.5 based on temperate and subtropical templates to establish the upper limit of waterlogging tolerance.

Cold stress (CS): Cold stress represented the low-temperature accumulation threshold limiting winter survival, which was determined by the cold stress temperature threshold (TTCS) and accumulation rate (THCS). Although *P. tsushimanus* overwinters inside the host wood (which provides partial thermal insulation), extreme winter cold remains a critical limiting factor in its northern distribution. Its native range in East Asia (specifically Tsushima Island, Japan) experiences mild winters, while its introduced range in East China (such as Shanghai and Jiangsu) experiences occasional winter temperatures reaching –10 °C [1,2,3]. To represent this distribution pattern, TTCS was set at –10 °C and THCS was set at −0.001 week^−1^, ensuring that the simulated distribution would match the known northern boundary of its survival.

Heat stress (HS): HS was defined by the heat stress temperature threshold (TTHS) and its accumulation rate (THHS). As heat stress begins to accumulate after temperatures exceed the upper developmental threshold, the TTHS must be ≥DV3. To restrict the potential distribution of *P. tsushimanus* in arid and extremely hot desert regions where its host *C. camphora* cannot survive, TTHS was set at 38 °C with a THHS of 0.005 week^−1^, which aligned with the climatic limits of subtropical evergreen broadleaf forests.

Dry stress (DS): DS was defined by the dry stress threshold (SMDS) and its accumulation rate (HDS). Because *P. tsushimanus* is highly sensitive to dry conditions, SMDS was set at 0.1 (equal to SM0). Through iterative model calibration, HDS was set at −0.005 week^−1^ to prevent the model from projecting potential establishment in arid regions of Northwest China and Central Asia, which is consistent with the moisture requirements of its host plant [7,33]. This dry stress setting is biologically congruent with the physiological vulnerability of *P. tsushimanus* across different developmental stages; drought-induced host desiccation is highly lethal to the moisture-sensitive egg stage and young, early-instar larvae residing in the phloem, as they lack robust sclerotization and are highly susceptible to water loss [2,27].

Wet stress (WS): WS was determined by the wet stress threshold (SMWS) and accumulation rate (HWS). In contrast to desert-adapted species, *P. tsushimanus* is highly adapted to humid subtropical environments. Therefore, the SMWS was set to a relatively high threshold of 2.5, with a low accumulation rate (HWS) of 0.002 week^−1^, enabling the insect to tolerate high-rainfall monsoonal climates in East and Southeast Asia without experiencing excessive wet stress accumulation.

Degree days per generation (PDD): PDD represents the thermal accumulation (degree days above DV0) required to complete the development from egg to adult emergence. According to the developmental biology of *P. tsushimanus* reported by Jia et al., the effective accumulated temperatures for the egg, larval, and pupal stages were 221.21, 955.69, and 206.00 °C·days, respectively. Summing these values resulted in a pre-adult developmental requirement of 1382.90 °C·days; consequently, the PDD was set to 1382.90 °C·days [27]. While the individual immature stages exhibit distinct lower developmental thresholds (egg: 9.03 °C, larva: 13.04 °C, pupa: 10.32 °C), utilizing the highest developmental threshold (larval threshold of 13.04 °C) as the uniform baseline (DV0) represents a biologically realistic, conservative bottleneck, as complete lifecycle development is physiologically limited by this highest threshold under cold conditions. Because the larval stage accounts for the vast majority (~70%) of both the generation time and the total thermal requirement of *P. tsushimanus*, aggregating these developmental constants above the larval threshold provides a robust, ecologically conservative representation of the species’ overall developmental requirements in the field [25].

Diapause index (DI): Although *P. tsushimanus* overwinters as larvae or adults in temperate regions, this behavior is facultative dormancy triggered directly by low winter temperatures rather than an obligate diapause mechanism. Consequently, the diapause index (DI) was not parameterized in the model [2].

#### 2.4.4. Classification of Ecoclimatic Index (EI) Values

To facilitate a consistent and direct comparative analysis, the prediction layers of *P. tsushimanus* and its host, *C. camphora*, and the EI values were categorized into five distinct suitability tiers. This classification scheme was designed to correspond to the five-level suitability framework employed in the RF model (unsuitable, very low, low, medium, and high). Specifically, an EI value of zero was defined as “unsuitable” for pest survival. The positive EI spectrum was then divided into four classes based on the species’ ecological tolerance and distribution records: very low (0 < EI ≤ 10), low (10 < EI ≤ 20), medium (20 < EI ≤ 30), and high (EI > 30) suitability. This harmonized classification enabled a spatially explicit evaluation of the overlap between the host and pest.

### 2.5. Potential Distribution of P. tsushimanus Constrained by Host Plant Distribution

To evaluate the potential distribution of *P. tsushimanus* under the ecological constraints of its host plant, the RF-predicted distribution of *C. camphora* and CLIMEX projected climatic suitability of the pest were integrated using ArcGIS. First, a suitability map of the host plant was converted into a binary raster. Grid cells corresponding to suitable habitats (incorporating very low, low, medium, and high suitability classes with values greater than 0) were reclassified to a value of 1, whereas unsuitable cells (with a value of 0) were assigned a value of 0. Subsequently, this binary host distribution map was used as a spatial mask to extract the potential distribution of *P. tsushimanus* simulated by the CLIMEX model [15]. Regions that were climatically suitable for the pest but lacked host availability were excluded, yielding a refined potential distribution map for *P. tsushimanus* that explicitly accounted for host plant constraints.

## 3. Results

### 3.1. Global Potential Distribution for the Host C. camphora Projected Using RF

#### 3.1.1. Model Performance and Key Environmental Factors

Table 3 presents the validation metrics (AUC, kappa, and TSS) used to evaluate the predictive accuracy of the RF model for *C. camphora* under contemporary climate conditions. The validation results indicated a high level of predictive performance and model robustness, with an AUC of 0.9535, a kappa coefficient of 0.8842, and a TSS of 0.8750. These evaluated metrics substantially exceeded their respective high-performance threshold criteria (AUC ≥ 0.90, kappa ≥ 0.85, and TSS ≥ 0.85), indicating that the RF model exhibited high reliability and was highly suitable for simulating the geographic distribution of *C. camphora*.

**Figure 2:**
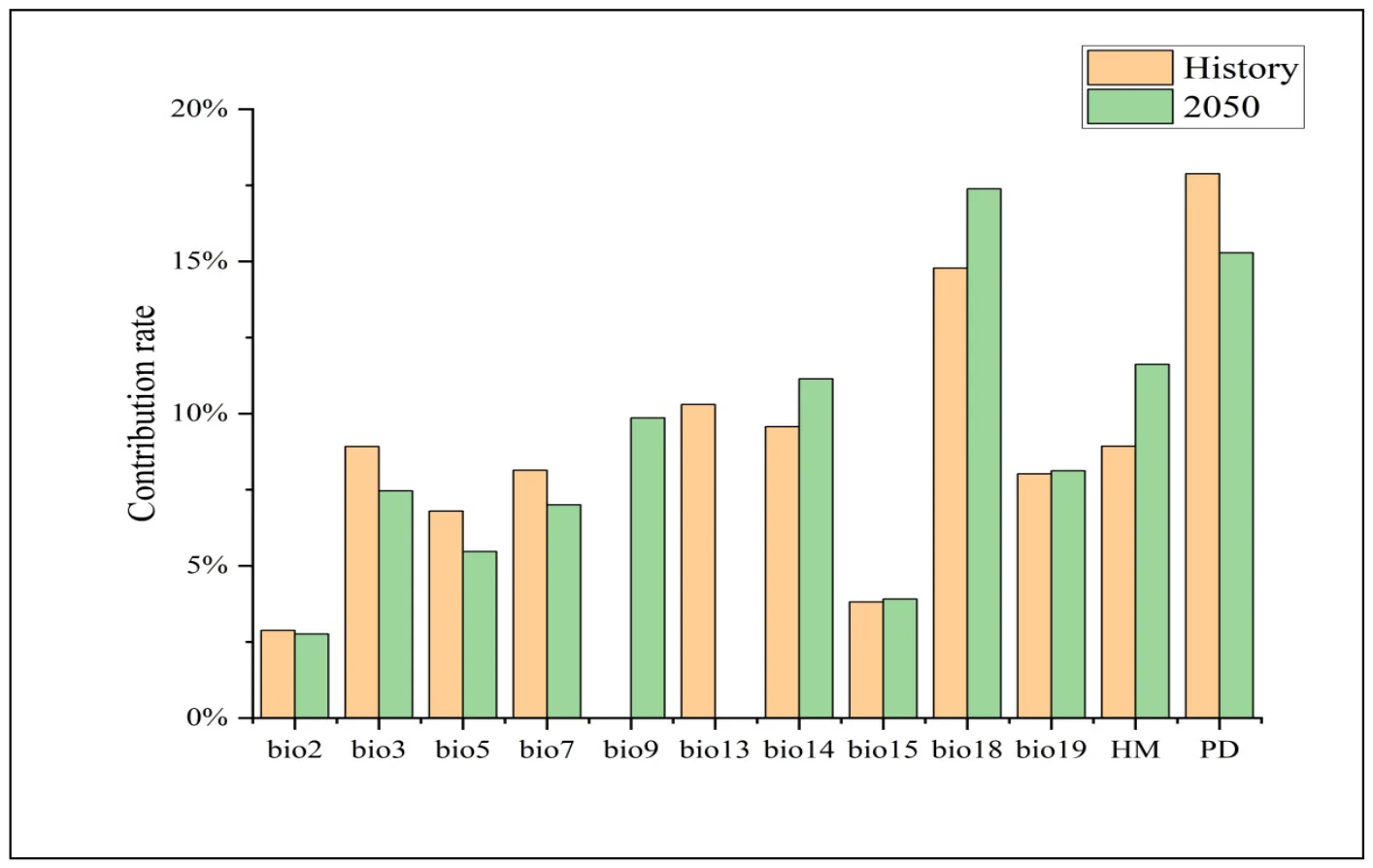
Relative contributions of key environmental variables influencing the potential distribution of *C. camphora*.

The relative contribution rates of the major environmental variables to the model outputs under different climate scenarios are shown in Figure 2. Under historical climatic conditions, the top five dominant variables driving the model were Population Density (PD; 17.88%), Precipitation of Warmest Quarter (BIO18; 14.78%), Precipitation of Wettest Month (BIO13; 10.30%), Precipitation of Driest Month (BIO14; 9.57%), and gHM (8.93%). Under the future climate scenario (2050), the ranking of variable importance shifted, with the top five contributors being: BIO18 (17.38%), PD (15.28%), gHM (11.62%), BIO14 (11.14%), and Mean Temperature of Driest Quarter (BIO9, 9.86%).

These results suggest that anthropogenic factors (specifically population density [PD] and Global Human Modification [gHM]) and climate-related moisture constraints (BIO18 and BIO14) consistently act as the primary drivers regulating the potential distribution of *C. camphora*. The prominent influence of PD and gHM in both eras indicated that the spatial pattern of *C. camphora* was heavily influenced by human activities and urban landscaping preferences. The increased influence of BIO18 coupled with the emergence of dry-quarter temperature constraints (BIO9) in the future indicated that *C. camphora* may experience increased sensitivity to seasonal water deficits under future climate warming.

#### 3.1.2. Potential Global Distribution Under the Historical Climate Scenario

**Figure 3:**
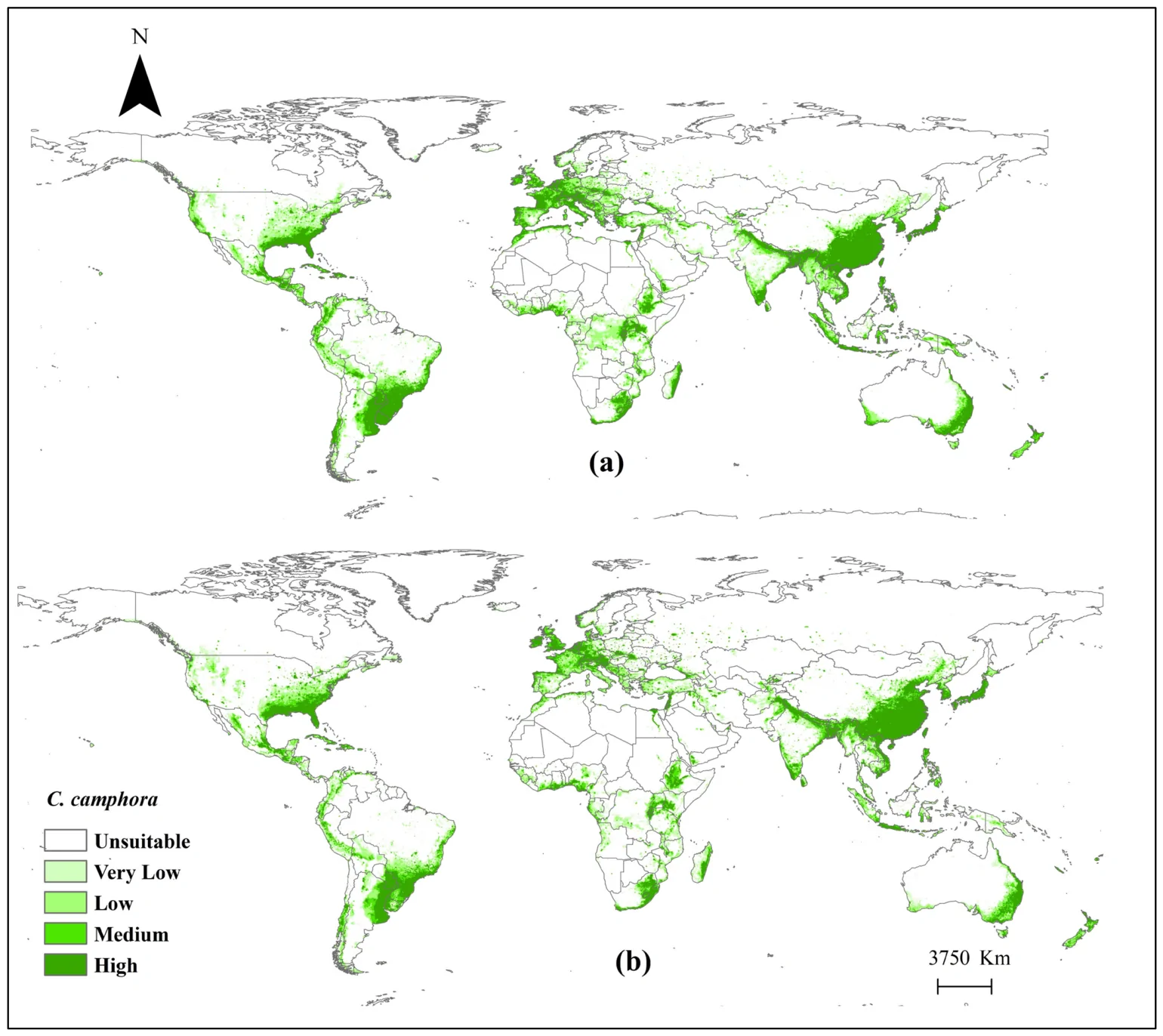
Projected potential global distribution of *C. camphora* under (**a**) historical and (**b**) future (SSP370) climate scenarios.

As shown in Figure 3a, the potential distribution of *C. camphora* exhibited distinct geographic and latitudinal gradients under historical climate scenarios (File S1). Core habitats classified as medium-to-high suitability were primarily concentrated in warm-temperate and humid subtropical regions. These highly suitable habitats were broadly and densely distributed across Southern and Eastern China, particularly in the Sichuan Basin, Yunnan, Guizhou, Hunan, Jiangxi, Guangdong, Fujian, Zhejiang, and Taiwan. This range extended northward to southern Japan and South Korea, closely aligning with the recognized native distribution of *C. camphora*. In North America, highly suitable regions were prominent in the southeastern United States, extending from eastern Texas across the Gulf Coast to the Florida Peninsula, and northward along the Atlantic coast to North Carolina. In South America, the core habitats were concentrated in the southeastern portion of the continent, specifically in southern Brazil (including Rio Grande do Sul, Santa Catarina, and Paraná), Uruguay, and northeastern Argentina. In Africa, high to medium suitability was primarily observed along the southeastern coastal belt, eastern South Africa, and parts of Madagascar. In Oceania, core habitats were situated along the eastern and southeastern coasts of Australia (primarily Queensland and New South Wales) and across New Zealand. In Europe, suitable habitats were more fragmented, with low-to-medium suitability largely restricted to coastal Western Europe and the Mediterranean Basin, including Portugal, Spain, Italy, and Greece.

#### 3.1.3. Potential Global Distribution Under the Future Climate Scenario

Under the future climate scenario (SSP370 for the year 2050; Figure 3b), the potential distribution of *C. camphora* exhibited a notable poleward expansion (File S2) (northward in the Northern Hemisphere and southward in the Southern Hemisphere), along with an overall increase in suitability levels. In East Asia, the northern boundary of suitable habitats in China shifted northward, encroaching deeper into Central and Northern China (Henan, Shandong, and Shaanxi provinces). In North America, the suitable range in the eastern United States expanded northward into the Midwest and Mid-Atlantic regions, and the intensity of suitability increased across the Southeast. In Europe, this model projected a notable expansion of suitable habitats. Areas of low to medium suitability expanded significantly from the Mediterranean coast into Western and Central Europe, including major portions of France, Germany, the United Kingdom, and Ireland, with high suitability zones emerging in Western Europe. Simultaneous poleward expansion was observed in the Southern Hemisphere. Highly suitable habitats expanded southward in southern Brazil, Uruguay, and northern Argentina. Similarly, suitable areas increased along the southern coast of Australia and expanded to the southern islands of New Zealand. This widespread geographic shift suggests that increasing global temperatures and altered precipitation patterns under future climate scenarios will likely alleviate low-temperature limitations in higher-latitude temperate zones, transforming these regions into climatically favorable habitats for *C. camphora*.

### 3.2. Potential Global Distribution of P. tsushimanus Projected Using CLIMEX

#### 3.2.1. Model Performance

**Figure 4:**
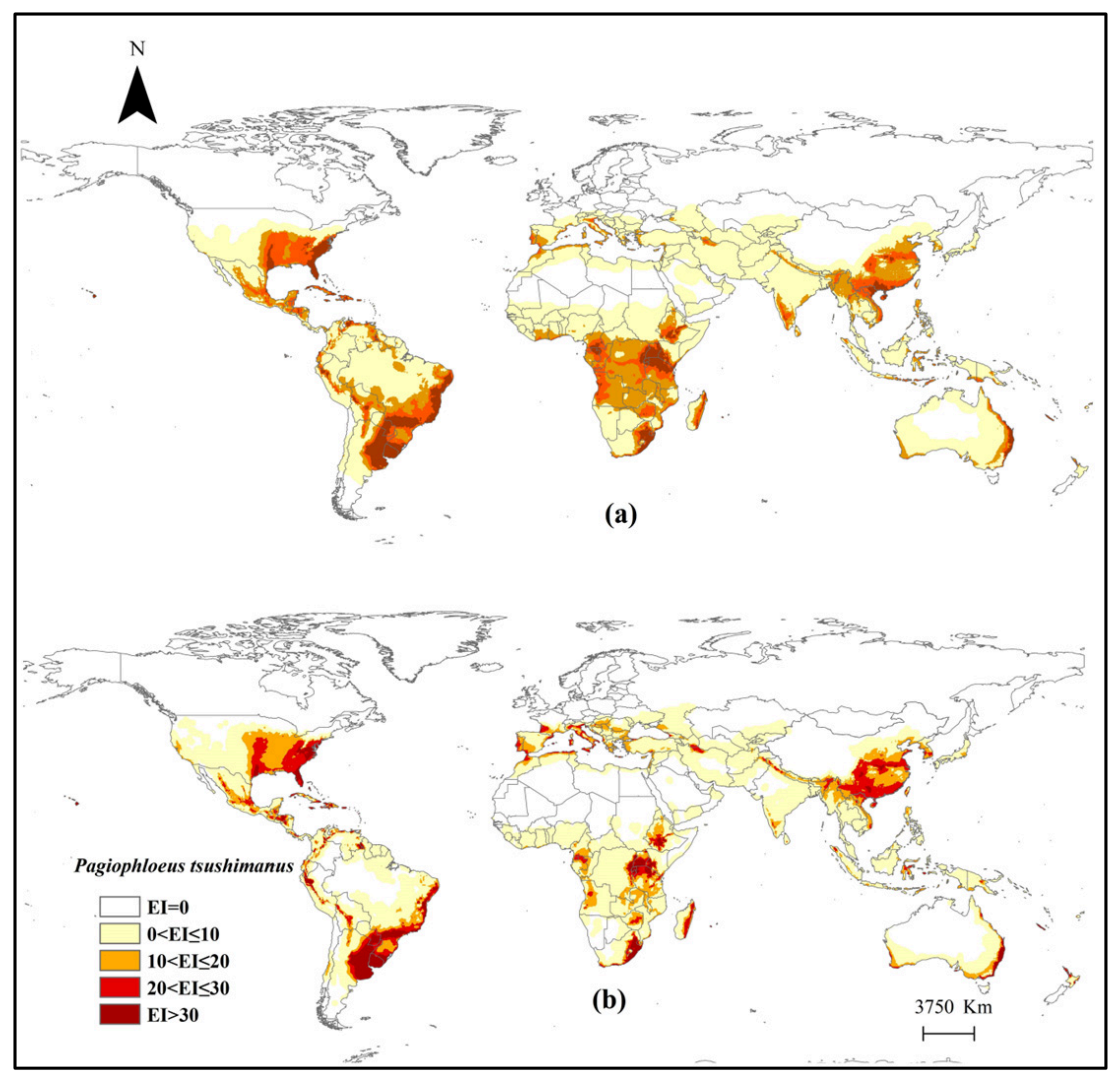
Potential global climatic suitability of *P. tsushimanus* projected by CLIMEX under (**a**) historical and (**b**) future climate scenarios.

An essential step in evaluating the robustness of the calibrated CLIMEX parameters was to validate the spatial agreement between the projected suitability zones and the known global occurrence records of *P. tsushimanus*. The simulation results demonstrated a high level of consistency, with all documented distribution records (primarily located in Japan and East China) falling within the climatically suitable areas projected by the model. Regions with higher historical infestation densities and established populations, such as East Asia, corresponded closely with high EI values (Figure 4a). This spatial congruence indicates that the calibrated parameter set was highly reliable and biologically representative of the climatic tolerance of the species.

#### 3.2.2. Potential Global Distribution Under the Historical Climate Scenario

Under the historical climate scenario, the CLIMEX model projected a widespread potential distribution of *P. tsushimanus* across warm temperate and subtropical regions, whereas cold high-latitude zones and hyperarid deserts remained unsuitable (Figure 4a). Highly suitable zones were heavily concentrated in East Asia, including Southern and Eastern China (the Yangtze River Basin and southern provinces), Southern Japan (the native range of the species), South Korea, and northern Vietnam. In North America, highly suitable zones comprised the southeastern United States, notably Florida, Georgia, Alabama, Mississippi, Louisiana, and the coastal Carolinas. In South America, the model projected extensive and highly suitable areas across southeastern Brazil, Uruguay, and northeastern Argentina (the La Plata Basin). In Africa, highly suitable habitats were projected across the central equatorial belt (the Congo Basin) and Madagascar, indicating warm and humid climates. In Europe, suitable areas were predominantly restricted to the Mediterranean coast (Spain, Italy, and Greece), where the model projected very low to low suitability. In Oceania, suitable areas were limited to the eastern coast of Australia (Queensland and New South Wales), showing marginal to low suitability.

#### 3.2.3. Potential Global Distribution Under the Future Climate Scenario

Under the future climate scenario (2050/A1B; Figure 4b), the potential distribution of *P. tsushimanus* exhibited an evident poleward shift and expansion, similar to the pattern projected for its host plant. In Asia, the overall northward migration of climatically suitable areas was projected. The core high-suitability zones in East Asia expanded significantly northward in China, extending beyond the Yangtze River into Central and Northern China (Henan, Shandong, and Shaanxi provinces). In North America, suitable zones expanded further northeastward and northwestward in the United States, with several mid-Atlantic and northeastern states emerging as new highly suitable areas. In South America, high-suitability zones in southern Brazil, Uruguay, and northern Argentina consolidated and expanded further south. In Europe, a pronounced northward expansion of suitable areas was projected. Suitable habitats extended from the Mediterranean Basin deep into Western and Central Europe, including France, Germany, and the United Kingdom, with certain areas in Southern Europe transitioning to medium and high suitability. In Africa, highly suitable areas south of the Saharan Desert exhibited noticeable fragmentation and a reduction in core suitability, particularly within the Congo Basin, although suitability in parts of southern Africa increased. In Oceania, climatically suitable zones along the southeastern coast of Australia and New Zealand expanded moderately, showing higher suitability values than in the historical scenario.

### 3.3. Potential Global Distribution of P. tsushimanus Constrained by Host Plant Distribution

#### 3.3.1. Potential Global Distribution Under the Historical Climate Scenario

**Figure 5:**
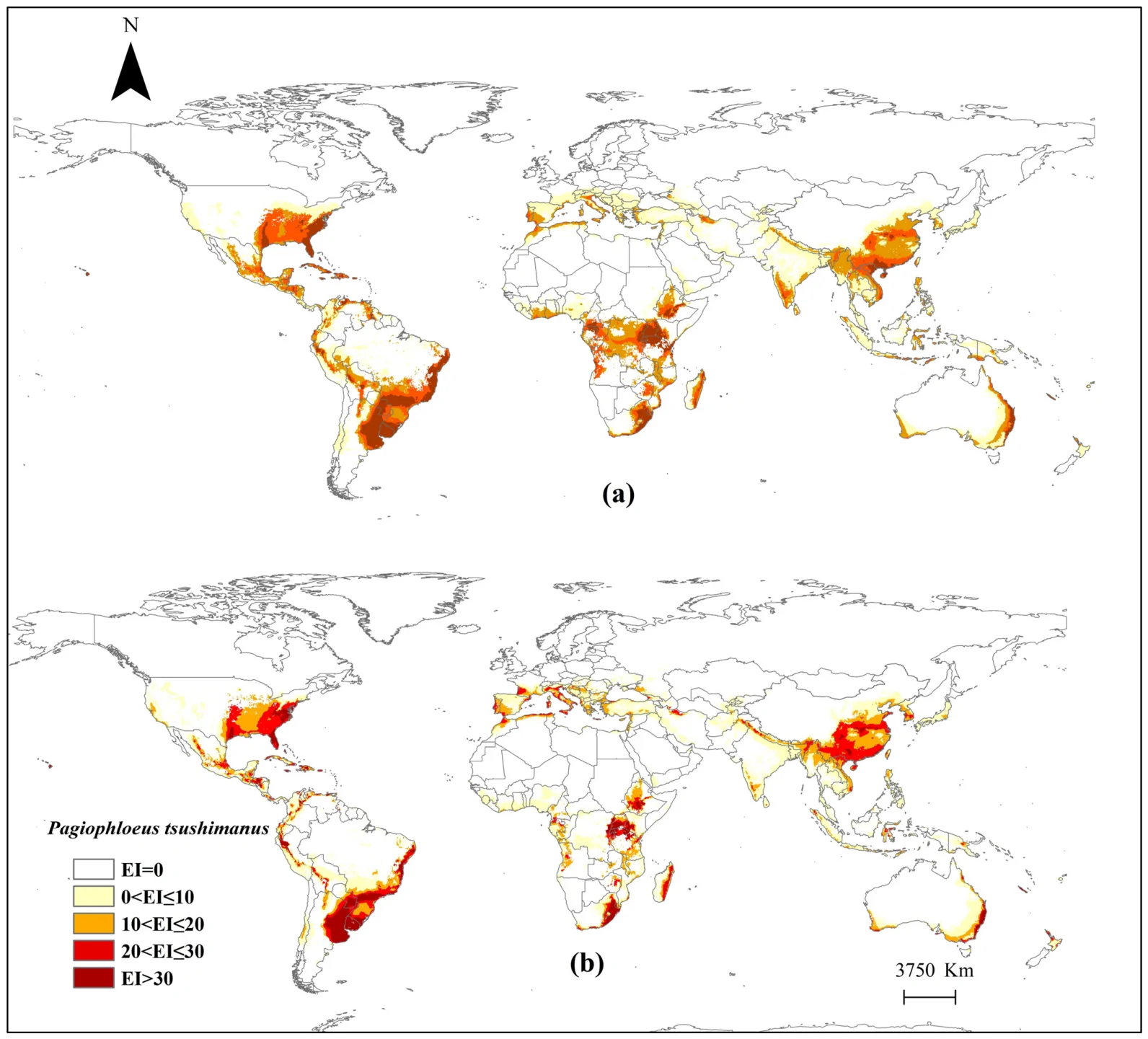
Host-constrained potential global distribution of *P. tsushimanus* under (**a**) historical and (**b**) future climate scenarios.

When the RF-predicted distribution of the host plant was applied as a spatial constraint over the CLIMEX climatic suitability projections, the projected potential global distribution of *P. tsushimanus* was significantly refined and restricted (Figure 5a). Compared to the unconstrained climatic suitability map, vast regions that were climatically favorable for the pest but lacked host plant availability, such as the Amazon Basin in South America and the Congo Basin in Central Africa, were successfully classified as unsuitable. Under the historical climate scenario, the potential distribution of *P. tsushimanus* was strictly confined to areas where both suitable climate and host resources coexisted. In Asia, suitable areas were tightly bound to the native and cultivated ranges of *C. camphora* and were primarily concentrated in Southern and Eastern China, Southern Japan, and South Korea, where high suitability was dominant. In North America, the potential range of *P. tsushimanus* was restricted to the southeastern United States (spanning from eastern Texas to Florida and North Carolina) and coastal California, where the host tree was either naturalized or planted. In South America, the highly suitable habitats were strictly localized in southeastern South America, specifically in southern Brazil, Uruguay, and northeastern Argentina, whereas the non-host Amazon Basin was completely excluded. In Europe, fragmented suitable zones were restricted to coastal Western Europe and the Mediterranean Basin. Suitable areas in Africa were highly restricted to southeastern Africa (portions of South Africa and Mozambique) and Madagascar. In Oceania, suitable habitats were located along the southeastern and eastern coasts of Australia and across parts of New Zealand.

#### 3.3.2. Potential Global Distribution Under the Future Climate Scenario

Under the future climate scenario (Figure 5b), the host-constrained potential distribution of *P. tsushimanus* exhibited an evident poleward shift driven by the spatial convergence of both host availability and climatic suitability. In Asia, suitable areas in China expanded significantly northward, with high-suitability zones extending beyond the Yangtze River into Central and Northern China, including Henan, Shandong, and southern Shaanxi provinces, tracking the projected range expansion of *C. camphora*. In North America, high-suitability regions shifted northeastward in the United States, expanding into the mid-Atlantic and northeastern states, while suitability along the Gulf Coast remained robust. In South America, suitable areas in southern Brazil, Uruguay, and northern Argentina consolidated and expanded further southward, showing a significant increase in high-suitability zones. In Europe, suitable climatic areas shifted and expanded northward from the Mediterranean rim to Western and Central Europe (France, Germany, and the United Kingdom), with several Western European countries transitioning to medium and high suitability. In Africa, suitable zones remained highly restricted and fragmented, with only a minor expansion in southeastern South Africa. In Oceania, suitable areas along the southeastern coast of Australia and New Zealand expanded moderately, showing higher suitability values than in the historical scenario. Overall, the integration of host plant constraints revealed a more realistic and ecologically sound future risk of invasion. Although climate change climatically unlocks high-latitude regions, the actual establishment and spread of *P. tsushimanus* remain strictly bound by future range shifts in its host, *C. camphora*.

#### 3.3.3. Distribution Area of Different Suitability Levels

**Figure 6:**
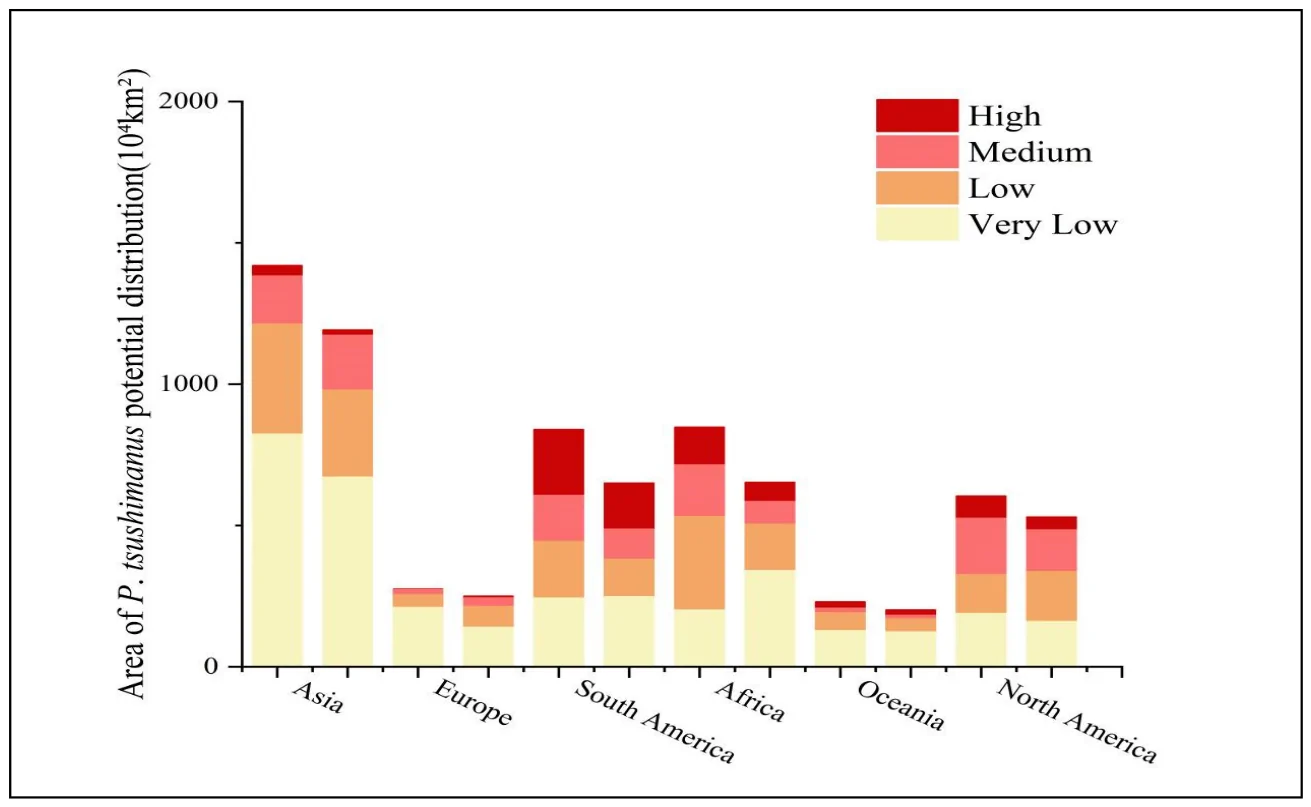
Potential suitable habitat area of *P. tsushimanus* across different continents under historical and future climate scenarios.

The predicted potentially suitable habitat areas of *P. tsushimanus* across different suitability levels under both historical and future climate scenarios are presented in Table 4, and their continental distributions are shown in Figure 6. Under historical climatic conditions, the total projected suitable habitat area for *P. tsushimanus* was 4238.03 × 10^4^ km^2^. This suitable area comprised 1828.49 × 10^4^ km^2^ of very low suitability, 1172.86 × 10^4^ km^2^ of low suitability, 751.04 × 10^4^ km^2^ of medium suitability, and 485.64 × 10^4^ km^2^ of high suitability habitats. Under the future SSP370 scenario for the 2050s, the total suitable area was projected to contract to 3475.79 × 10^4^ km^2^, representing a net reduction of 17.99%. A detailed level-by-level comparison revealed that all suitability categories had declined, with the highest-suitability habitats experiencing the most severe contractions. Specifically, very low suitable habitats decreased slightly by 6.50% (to 1709.71 × 10^4^ km^2^). Low- and medium-suitability habitats decreased by 23.07% (to 902.29 × 10^4^ km^2^) and 23.75% (to 572.63 × 10^4^ km^2^), respectively. Notably, the high-suitability category experienced the most substantial reduction, decreasing by 39.22% from 485.64 × 10^4^ km^2^ to only 291.16 × 10^4^ km^2^. This disproportionate contraction indicates the global degradation of optimal habitats for *P. tsushimanus* under future high-emission scenarios.

On the continental scale, suitable habitats were unevenly distributed; however, they exhibited a consistent declining trend across all regions (Figure 6). Asia had the largest potentially suitable area in both scenarios, with its total suitable habitat decreasing from approximately 1400 × 10^4^ km^2^ to approximately 1200 × 10^4^ km^2^ in the 2050s, along with a substantial reduction in the high-suitability category. South America and Africa also contained extensive suitable areas, both decreasing from approximately 850 × 10^4^ km^2^ under historical conditions to 650 × 10^4^ km^2^ by the 2050s. North America had a moderately suitable area, decreasing from approximately 600 × 10^4^ km^2^ to 550 × 10^4^ km^2^. Europe (approximately 300 × 10^4^ to 270 × 10^4^ km^2^) and Oceania (approximately 250 × 10^4^ to 200 × 10^4^ km^2^) had smaller suitable envelopes but followed the same declining trajectory. Collectively, these results demonstrate a global-scale habitat contraction for *P. tsushimanus* under future scenarios, primarily driven by a reduction in highly suitable core zones.

### 3.4. Distribution Shifts of P. tsushimanus and Its Host C. camphora Under Climate Change

**Figure 7:**
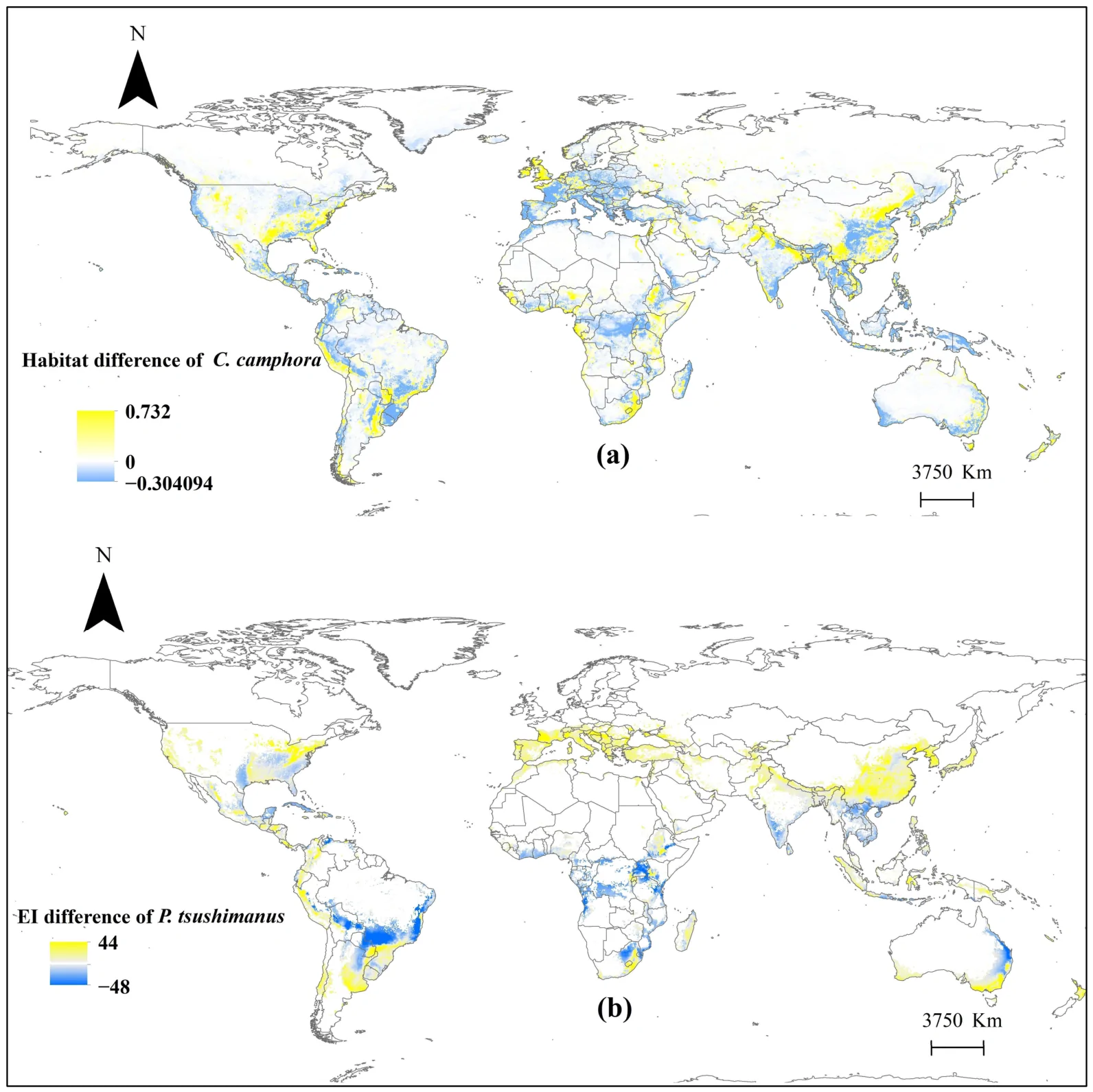
Spatial shift and suitability difference (future scenario minus historical scenario) of (**a**) *C. camphora* habitat suitability and (**b**) *P. tsushimanus* Ecoclimatic Index (EI) under climate change.

To visually quantify the spatial expansion and contraction of suitable habitats driven by climate change, spatial difference maps (future scenario minus historical scenario) were generated for both *C. camphora* and *P. tsushimanus* (Figure 7). For the host plant, *C. camphora*, the habitat suitability difference ranged from −0.304 to 0.732 (Figure 7a). Pronounced suitability increases were prominently observed in higher-latitude regions, particularly in Central and Northern China (spreading north of the Yangtze River Basin), Western and Central Europe (including France, Germany, and the United Kingdom), the northeastern United States, and New Zealand. Conversely, minor suitability declines were restricted to certain low-latitude subtropical areas, such as parts of Southern China and central Brazil. This pattern suggests a clear poleward shift in the host ecological niche.

For the pest, *P. tsushimanus*, under host plant constraints, the EI difference ranged from −48 to 44 (Figure 7b). Highly consistent with its host, the pest exhibited substantial suitability increases in Central and Eastern China, the northeastern United States, and southeastern South America (southern Brazil and Uruguay). However, pronounced suitability declines were observed in low-latitude regions, particularly in Southeast Asia, the low-latitude zones of Southern China, and central-southern Brazil.

This comparative analysis reveals a high degree of spatial convergence between the host and pest. Under future climate change conditions, although the northern margins of both species will expand owing to the relaxation of cold limitations, their southern boundaries will contract, likely owing to extreme summer heat stress and associated moisture deficits. Consequently, the threat posed by *P. tsushimanus* is projected to shift from the traditional tropical/subtropical zones to newly suitable temperate regions, tracking host migration.

## 4. Discussion

### 4.1. Ecological Drivers of Host Distribution and the Influence of Anthropogenic Factors

As a strictly monophagous herbivore, the potential geographic range of *P. tsushimanus* is fundamentally linked to the spatial availability of its host plant, *C. camphora*. Consequently, the bioclimatic and anthropogenic drivers regulating host suitability represent de facto indirect constraints on pest establishment and invasion risks. Our RF model demonstrated that *C. camphora* distribution was primarily co-regulated by moisture-related predictors (BIO18 and BIO14) and human activity variables (PD and gHM). From a hydrological perspective, because *C. camphora* is highly sensitive to warm-season water deficits, seasonal droughts that induce host xylem cavitation and subsequent forest decline could potentially restrict *P. tsushimanus* population growth due to host deprivation. While direct evidence of host-mediated weevil mortality remains to be empirically tested, host physiological stress under drought is likely a critical limiting factor for its realized distribution [54]. This indirect climatic constraint explains the projected suitability degradation of this pest in traditional subtropical zones, where future heat and drought stresses are expected to compromise host health.

More importantly, the prominent contributions of anthropogenic variables, specifically population density (PD; 17.88% in the historical scenario and 15.28% in 2050) and Global Human Modification (gHM; 8.93% in the historical scenario and 11.62% in 2050), indicate that human activities serve as artificial facilitators of *P. tsushimanus* invasion. As *C. camphora* is extensively planted for urban greening and roadside landscaping, its spatial pattern closely tracks human population density (PD) and Global Human Modification (gHM) [7,8]. In these human-dominated biomes, active horticultural management, such as intensive irrigation, buffers host trees against natural climatic stressors, enabling them to thrive in otherwise marginal regions. These artificially sustained urban camphor forests hypothetically act as favorable spatial corridors and microclimatic refuges, bypassing natural dispersal barriers and facilitating the rapid establishment, population expansion, and long-distance spread of *P. tsushimanus* [55]. In addition, while climate warming is projected to shift the climatic envelope of *C. camphora* northward, its actual range expansion faces critical non-climatic and socioeconomic bottlenecks. On the one hand, rising global temperatures are projected to increase aridity across many tropical and subtropical zones, potentially compounding moisture stress and making these areas less favorable for this moisture-dependent tree. On the other hand, in newly suitable higher-latitude northern regions, land allocation represents a major socio-ecological barrier. Landowners in these highly productive temperate areas typically prioritize high-value traditional agricultural crops over forestry or landscape trees like *C. camphora*. Consequently, despite expanded climatic suitability, the actual transplanting and establishment of *C. camphora* in northern regions will likely be heavily constrained by agricultural land-use competition and economic decisions.

### 4.2. Distribution Shifts in P. tsushimanus and Its Host C. camphora Under Climate Change

Under the future climate scenario, both *P. tsushimanus* and *C. camphora* exhibited highly convergent poleward shifts and expansions. Notably, we identified new suitable invasion hotspots in Central and Northern China (Shandong, Henan, and Shaanxi), Western and Central Europe (including France, Germany, and the United Kingdom), and the northeastern United States. These regions, historically protected by low-temperature limitations, will experience an alleviation of cold stress under future warming trajectories, revealing previously unsuitable environments for both the host and pest.

This shift in distribution is consistent with the physiological tolerance parameters of *P. tsushimanus*. In terms of thermal requirements, the lower developmental threshold of the pest is 13.04 °C, with an optimal temperature range of 20–28 °C and an upper temperature threshold of 33 °C; completion of its life cycle requires a cumulative 1382.90 °C·days of effective degree-days above DV0 [27]. In high-latitude regions such as Northern China and Western Europe, historical winter temperatures frequently decreased below the cold stress threshold (−10 °C), and annual accumulated temperature did not meet the degree-day requirement for population establishment. Under future warming conditions, the alleviation of cold stress and increased thermal accumulation will enable these regions to satisfy the basic developmental and overwintering conditions of the pest, thereby forming new suitable habitats. Furthermore, the ecological response of *P. tsushimanus* to climate change is intimately coupled with its life-cycle characteristics. As a strictly monovoltine species, rising temperatures under future climate warming are unlikely to increase the annual generation number of *P. tsushimanus*; however, they will significantly alter its seasonal phenology [2,27]. Warming temperatures are projected to advance the onset of spring activity and delay winter dormancy, thereby extending the overall duration of the active growing season. While this prolonged activity window may increase cumulative feeding damage, elevated summer temperatures may also induce summer diapause [2,27]. Additionally, climate warming is expected to modify the overwintering population structure by shifting the ratio of overwintering larvae to adults, particularly by increasing the proportion of older, more cold-hardy larvae, which could enhance winter survival and accelerate spring emergence.

Table 4 presents a net global contraction of the total suitable area of the pest of 17.99%, with the most optimal (high suitability) habitats experiencing a substantial reduction of 39.22% under the SSP370 scenario. This contrasting trend suggests that, although climate change alleviates cold constraints at high latitudes, it simultaneously exacerbates thermal and drought stress in traditional low-latitude subtropical core zones [13,14]. From the modeling perspective, when temperatures exceed the calibrated heat-stress threshold of 38 °C and soil moisture decreases below the dry-stress threshold of 0.1, the predicted climatic suitability of *P. tsushimanus* declines drastically [25]. While these thresholds are aligned with the empirical developmental limits documented in laboratory rearing studies [27], they represent model-calibrated parameters for population growth rather than experimentally validated physiological lethal limits. The projected degradation of suitability in Southeast Asia, Southern China, and central-southern Brazil indicates that increasing extreme temperatures and seasonal precipitation deficits may exceed the physiological thermal and moisture limits of *P. tsushimanus* and its host, leading to habitat fragmentation in their historical ranges.

Additionally, the ecological interaction between *P. tsushimanus* feeding behavior and plant pathogens represents a critical, yet overlooked, biosecurity risk under climate change. Larvae boring extensive galleries through the phloem and xylem directly disrupt hydraulic transport, while adults overwintering in branch forks in facultative diapause emerge to feed on branch bark and buds in early spring, further compromising host vigor [2,27]. The physical wounds inflicted by both larvae and adults serve as major entry pathways that facilitate the penetration of potential wood pathogens into the host vascular system [16]. Under future warming scenarios, this risk of pathogen transmission is projected to amplify. Climatic shifts may disrupt the phenological synchrony between the timing of host bud break and adult post-overwintering feeding, potentially creating wider infection windows during highly susceptible growth stages of *C. camphora*. Simultaneously, warmer and more humid spring conditions can accelerate the development and replication cycles of potential plant pathogens, thereby compounding the overall decline of camphor tree plantations.

### 4.3. Methodological and Ecological Significance of Host Constraints

A major challenge in insect pest risk assessment using species distribution models (SDMs) is the “over-prediction” of suitable areas when biotic interactions are ignored [56]. Traditional climatic niche models such as unconstrained CLIMEX or MaxEnt projections typically predict extensive high-suitability zones for specialized pests in regions that satisfy their thermodynamic requirements but lack host plant resources [15]. In this study, the unconstrained CLIMEX projection for *P. tsushimanus* generated massive “false positive” suitability zones in the Amazon Basin of South America and the Congo Basin of Central Africa owing to their warm and humid tropical climates. However, because *P. tsushimanus* is a strictly monophagous pest with an obligate trophic dependence on *C. camphora*, the establishment of wild populations in these native tropical rainforests is ecologically impossible [5,6]. Using the RF-predicted distribution of *C. camphora* as a spatial constraint (mask), these biologically unrealistic suitability zones were successfully eliminated. The resulting host-constrained global potential distribution map represents a notable advancement in ecological realism. This trophic-constrained modeling framework highlights that biotic interactions (host availability) can be more restrictive than abiotic factors in defining the realized niches of specialized herbivores [15,56]. Consequently, incorporating host constraints is essential for preventing the overestimation of biosecurity risks and ensuring that monitoring and quarantine resources are directed toward areas with genuine exposure risks.

Host shift is a common phenomenon in biological invasions, and the trophic niche of invasive herbivores may expand during colonization [57]. Presently, *P. tsushimanus* has only been recorded feeding on *C. camphora* in both its native and invaded ranges, indicating a high degree of host specialization [5,6]. However, under future climate change and new invasion scenarios, there is potential for host range expansion: increased population pressure in degraded native habitats may drive the pest to exploit alternative host plants; in contrast, in newly invaded regions where *C. camphora* is scarce, populations may adapt to closely related species within the genus *Cinnamomum* or the family *Lauraceae* that are widely cultivated or naturally distributed [57]. If a host shift occurs, the actual potential distribution range of *P. tsushimanus* could be broader than that predicted in this study, and the corresponding biosecurity risk would also increase. Therefore, the results of this study represent a conservative risk assessment based on currently known host specificity, and long-term monitoring of the host adaptation of the pest in invaded areas is required.

### 4.4. Model Limitations and Future Perspectives

Although the integrated CLIMEX and RF modeling approach provides a robust and ecologically realistic prediction of the potential distribution of *P. tsushimanus*, certain methodological limitations must be acknowledged. The semi-mechanistic CLIMEX model primarily simulates geographical suitability based on macroclimatic inputs, assuming that species respond uniformly to ambient atmospheric parameters. However, as a wood-boring pest, the larvae of *P. tsushimanus* reside within the phloem and xylem of camphor trees. This cryptic lifestyle provides a major microclimatic buffering effect and insulates developmental stages against extreme ambient winter cold or summer heat waves [58]. Consequently, CLIMEX may underestimate the potential survival of the pest in marginal zones where microclimatic shelters exist. Additionally, CLIMEX does not incorporate biotic interactions such as natural enemy dynamics (parasitoids) or interspecific competition, which can restrict the niche of the pest in nature [56]. Furthermore, a critical source of uncertainty in our integrated projections stems from the discrepancy between the climate model generations and historical baseline periods used for the host and the pest. Specifically, the host plant *C. camphora* was modeled using the CMIP6 SSP3-7.0 scenario with a 1970–2000 baseline, whereas the pest *P. tsushimanus* was modeled using the CMIP3/AR4 SRES A1B scenario with a 1961–1990 baseline. This mismatch was dictated by data availability, as CLIMEX future simulations natively rely on the CliMond database, which is restricted to SRES or RCP formats. Although both SRES A1B and SSP3-7.0 represent comparable intermediate-to-high greenhouse gas emission pathways (both projecting a global mean temperature anomaly of approximately 1.5–2.2 °C by the mid-21st century), they belong to different GCM generations and utilize distinct baseline periods. Consequently, the spatial overlay of these suitability maps represents a geographical convergence under comparable thermal pathways rather than a perfectly synchronized, identical projection, and the combined outputs must be interpreted with caution regarding these underlying GCM variations.

Second, although highly accurate, the correlative RF model is fundamentally limited by its reliance on historical occurrence data and its lack of explicit physiological mechanisms [18,22]. While we applied spatial rarefaction to minimize spatial autocorrelation, our reliance on a simple random data-splitting strategy for model training and testing represents a limitation, as it may still carry residual spatial autocorrelation. Future work should incorporate spatial block cross-validation to prevent potential metric inflation and more rigorously evaluate model transferability across novel environments. Although we integrated key human variables, the necessity of treating the gHM as static under future projections introduces potential spatial uncertainties. A common challenge in future ecological forecasting is the lack of standardized global projection datasets for certain anthropogenic variables, such as gHM, under different future SSPs. To address this limitation, we treated the gHM as a static predictor in the 2050 projection. Human modification patterns will inevitably evolve by the mid-21st century, but a static baseline was used as a pragmatic approach when future high-resolution anthropogenic scenario data were unavailable [59]. Furthermore, our modeling framework assumes that *P. tsushimanus* will shift its range in spatial congruence with its host plant. In reality, the natural dispersal speed of the pest (regulated by adult flight capacity) is considerably slower than the rapid range expansion predicted under future climate change scenarios, indicating that long-distance range shifts will remain highly reliant on human-mediated passive transport [10]. Additionally, transferring species distribution models into future temporal scenarios inevitably introduces uncertainties regarding model transferability and mathematical extrapolation under novel climate conditions [18,22]. Although we utilized global occurrence records to fully represent the realized environmental niche of *C. camphora* and minimize extrapolation bias, future research incorporating multivariate environmental similarity surface (MESS) analysis and multi-model GCM ensembles would help further quantify these spatial uncertainties.

Future research should focus on addressing these gaps by incorporating high-resolution urban microclimate models to more accurately capture the temperature buffering in tree trunks. Additionally, integrating laboratory-determined thermal tolerances with multi-agent simulation models that account for natural insect dispersal rates and global timber trade pathways may result in more precise biosecurity forecasts.

### 4.5. Recommendations for Pest Management and Quarantine

The projected northward expansion of *P. tsushimanus* suitability under climate change, particularly in the newly suitable regions of Central and Northern China, Western Europe, and the northeastern United States, highlights the need for proactive quarantine and integrated pest management (IPM) strategies [11]. Our findings have critical implications for global forest biosecurity and quarantine policies. Forestry protection departments in newly suitable areas, particularly in Western Europe and Northern China, must implement proactive early warning programs. Because the cryptic, wood-boring life stages (eggs, larvae, and pupae) of *P. tsushimanus* are highly prone to passive long-distance dispersal through the movement of live nursery stock, strict phytosanitary inspections and quarantine measures must be enforced on imported *C. camphora* trees. This will prevent the accidental introduction and establishment of this pest in newly identified climatically suitable regions [60,61]. Because direct chemical or physical control of wood-boring weevils remains challenging following the establishment of populations, prevention and early detection are the most effective management options.

Strict quarantine regulations must be enforced at international ports and regional forestry borders with a focus on inspecting imported live *C. camphora* nursery stock and unmanufactured wood packaging materials that can easily harbor hidden eggs and larvae [60]. For newly identified, highly suitable regions, local forestry departments should establish active early warning monitoring programs using visual canopy inspections, trap trees, or potential future semiochemical attractants. To detect early signs of infestation, such as entry holes or frass [61]. Additionally, planting a diverse mix of tree species rather than monocultured camphor plantations in urban green spaces may reduce resource connectivity for *P. tsushimanus*, thereby mitigating the potential risk of widespread pest outbreaks [62].

## 5. Conclusions

In this study, we projected the potential global distribution of *P. tsushimanus* and its host *C. camphora* by integrating the Random Forest model and CLIMEX model, and analyzed their distribution shifts under future climate change. The results show that the potential distribution of the host *C. camphora* is primarily constrained by moisture-related factors, with precipitation deficit and Precipitation of Warmest Quarter (BIO18) as the dominant climatic drivers, while Global Human Modification (gHM) also exerts a strong influence as a key anthropogenic factor. Under current climatic conditions, the potential suitable habitat of *P. tsushimanus* (constrained by host availability) totals 4238.03 × 10^4^ km^2^, with core distribution areas concentrated in East Asia, the southeastern United States, southeastern South America, southeastern Africa and Madagascar, eastern coastal Australia, and the Mediterranean coast of Europe. Under future climate change, both *C. camphora* and *P. tsushimanus* are projected to shift poleward, unlocking new high-suitability invasion hotspots in higher-latitude temperate zones, specifically in Western and Central Europe, Northern China, and the Northeastern United States. Conversely, suitability in traditional subtropical core ranges is projected to degrade due to increased thermal and drought stresses, leading to a net global suitability contraction of 17.99%, with high-suitability habitats decreasing by 39.22%. These findings demonstrate that biotic interactions (host availability) and human modification are critical determinants of realized niche limits. The host-constrained maps generated in this study provide a realistic early-warning tool for global forest health. We recommend that forestry departments in newly suitable temperate zones implement strict phytosanitary inspections on imported *C. camphora* nursery stocks and establish active monitoring programs to prevent the accidental introduction and establishment of this cryptic, wood-boring pest.

## Figures and Tables

**Table 1:** Bioclimatic predictor variables used in the Random Forest modeling of *C. camphora*.

Variable Code	Variable Full Name (Unit)	Data Source
BIO1	Annual Mean Temperature (°C)	WorldClim 2.1
BIO2	Mean Diurnal Range (°C) *	
BIO3	Isothermality (-) *	
BIO4	Temperature Seasonality (°C)	
BIO5	Max Temperature of Warmest Month (°C) *	
BIO6	Min Temperature of Coldest Month (°C)	
BIO7	Temperature Annual Range (°C) *	
BIO8	Mean Temperature of Wettest Quarter (°C)	
BIO9	Mean Temperature of Driest Quarter (°C) *	
BIO10	Mean Temperature of Warmest Quarter (°C)	
BIO11	Mean Temperature of Coldest Quarter (°C)	
BIO12	Annual Precipitation (mm)	
BIO13	Precipitation of Wettest Month (mm)*	
BIO14	Precipitation of Driest Month (mm) *	
BIO15	Precipitation Seasonality (%) *	
BIO16	Precipitation of Wettest Quarter(mm)	
BIO17	Precipitation of Driest Quarter(mm)	
BIO18	Precipitation of Warmest Quarter(mm) *	
BIO19	Precipitation of Coldest Quarter (mm) *	
gHM	Global Human Modification (-) *	Conservation Science Partners
HF	Human Footprint (-)	Wildlife Conservation Society
PD	Population Density(persons/km^2^) *	WorldPop

Note: Variables with the highest relative importance for model prediction are marked with an asterisk *.

**Table 2:** CLIMEX parameter values for *P. tsushimanus*.

CLIMEX Parameter	Final Parameter Value
Temperature requirements	
DV0—Lower temperature threshold (°C)	13.04
DV1—Lower optimum temperature (°C)	20
DV2—Upper optimum temperature (°C)	28
DV3—Upper temperature threshold (°C)	33
Soil moisture	
SM0—Lower soil moisture threshold	0.1
SM1—Lower optimal soil moisture	0.6
SM2—Upper optimal soil moisture	0.8
SM3—Upper soil moisture threshold	1.5
Cold stress	
TTCS—Cold stress temperature threshold (°C)	−10
THCS—Cold stress temperature rate (week^−1^)	−0.001
Heat stress	
TTHS—Heat stress temperature threshold (°C)	38
THHS—Heat stress temperature rate (week^−1^)	0.005
Dry stress	
SMDS—Dry stress threshold	0.1
HDS—Dry stress rate (week^−1^)	−0.005
Wet stress	
SMWS—Wet stress threshold	2.5
HWS—Wet stress rate (week^−1^)	0.002
PDD—Positive degree-days (°C·days)	1382.90

**Table 3:** Evaluation indicators for the prediction accuracy of *C. camphora* by RF.

Target Species	Climate Scenario	Period	AUC *	Kappa *	TSS *
*C. camphora*	History	1970–2000	0.9535	0.8842	0.8750

* AUC, kappa, and TSS are 3 indicators used to evaluate model accuracy. Threshold values for discrimination abilities were AUC ≥ 0.90, kappa ≥ 0.85, and TSS ≥ 0.85.

**Table 4:** Area changes in different suitability levels of *P. tsushimanus*.

Decades Scenarios Predicted Area/10^4^ km^2^					
		Total very low	Total low	Total Medium	Total High
		suitable habitat	suitable habitat	suitable habitat	suitable habitat
History	—	1828.49	1172.86	751.04	485.64
2050s	SSP370	1709.71	902.29	572.63	291.16
Increase/decrease rate (%) [Compared to the current distribution]					
		Total very low	Total low	Total Medium	Total High
		suitable habitat	suitable habitat	suitable habitat	suitable habitat
2050s	SSP370	−6.50	−23.07	−23.75	−39.22

## Data Availability

The processed occurrence datasets (Tables S1–S3), the R analysis script (Script S1), and the final projected suitability raster layers (Files S1 and S2) supporting the conclusions of this article are fully available in the Supplementary Materials of this journal paper.

## Supplementary Materials

The following supporting information can be downloaded at: https://www.mdpi.com/article/10.3390/insects17101008/s1, Table S1: The coordinates of the *P. tsushimanus*; Table S2: The coordinates of *C. camphora* used for Random Forest modeling (1888 records); Table S3: The coordinates of *C. camphora* used for geographic visualization (300 records); Script S1: R script for the Random Forest modeling of *C. camphora*; File S1: *C. camphora* historical suitability; File S2: *C. camphora*_2050s_suitability.tif.

## Abbreviations

The following abbreviations are used in this manuscript:

*P. tsushimanus*	*Pagiophloeus tsushimanus*
*C. camphora*	*Cinnamomum camphora*
RF	Random Forests
gHM	Global Human Modification
HF	Human Footprint
PD	Population Density

## References

[1] Huang J., Wu S., Gao L., Yang L. (2014). Diagnosis and damage of weevil pest *Pagiophloeus tsushimanus* on camphor tree. J. Zhejiang A F Univ..

[2] Chen C., Zhang C.C., Li S.Y., Zhu H., Fan B.Q., Wang Y., Su P., Han Y.Y., Hao D.J. (2021). Biological traits and life history of *Pagiophloeus tsushimanus* (Coleoptera: Curculionidae), a weevil pest on camphor trees in China. J. For. Res..

[3] Morimoto K. (1982). The family Curculionidae of Japan. I. Subfamily Hylobiinae. Esakia.

[4] Chen C., Li S.Y., Zhu H., Fan B.Q., Wang Y., Hao D.J. (2020). Identification and evaluation of reference genes for gene expression analysis in the weevil pest *Pagiophloeus tsushimanus* using RT-qPCR. J. Asia-Pac. Entomol..

[5] Li S.Y., Li H., Chen C., Hao D.J. (2023). Tolerance to dietary linalool primarily involves co-expression of cytochrome P450s and cuticular proteins in *Pagiophloeus tsushimanus* (Coleoptera: Curculionidae) larvae using SMRT sequencing and RNA-seq. BMC Genom..

[6] Qiao H., Zhu H., Li H., Chen H.J., Li S.Y., Chen C., Hao D.J. (2023). Isolation and characterization of gut bacteria associated with the degradation of host-specific terpenoids in *Pagiophloeus tsushimanus* (Coleoptera: Curculionidae) larvae. J. Insect Sci..

[7] Li D., Lin H.Y., Wang X., Bi B., Gao Y., Shao L., Zhang R., Liang Y., Xia Y., Zhao Y.P. (2023). Genome and whole-genome resequencing of *Cinnamomum camphora* elucidate its dominance in subtropical urban landscapes. BMC Biol..

[8] Xiang Z., Zhao M., Ogbodo U.S. (2020). Accumulation of urban insect pests in China: 50 years’ observations on camphor tree (*Cinnamomum camphora*). Sustainability.

[9] Pureswaran D.S., Roques A., Battisti A. (2018). Forest insects and climate change. Curr. For. Rep..

[10] Meurisse N., Rassati D., Hurley B.P., Brockerhoff E.G., Haack R.A. (2019). Common pathways by which non-native forest insects move internationally and domestically. J. Pest Sci..

[11] Venette R.C., Kriticos D.J., Magarey R.D., Koch F.H., Baker R.H.A., Worner S.P., Gómez Raboteaux N.N., McKenney D.W., Dobesberger E.J., Yemshanov D. (2010). Pest risk maps for invasive alien species: A roadmap for improvement. BioScience.

[12] Pörtner H.O., Roberts D.C., Tignor M.M.B., Poloczanska E.S., Mintenbeck K., Alegría A., Craig M., Langsdorf S., Löschke S., Möller V. (2022). Climate Change 2022: Impacts, Adaptation and Vulnerability.

[13] Deutsch C.A., Tewksbury J.J., Huey R.B., Sheldon K.S., Ghalambor C.K., Haak D.C., Martin P.R. (2008). Impacts of climate warming on terrestrial ectotherms across latitude. Proc. Natl. Acad. Sci. USA.

[14] Lehmann P., Ammunét T., Barton M., Battisti A., Eigenbrode S.D., Jepsen J.U., Kalinkat G., Neuvonen S., Niemelä P., Terblanche J.S. (2020). Complex responses of global insect pests to climate warming. Front. Ecol. Environ..

[15] Liebhold A.M., McCullough D.G., Blackburn L.M., Frankel S.J., Von Holle B., Aukema J.E. (2013). A highly aggregated geographical distribution of forest pest invasions in the USA. Divers. Distrib..

[16] Ramsfield T.D., Bentz B.J., Faccoli M., Jactel H., Brockerhoff E.G. (2016). Forest health in a changing world: Effects of globalization and climate change on forest insect and pathogen impacts. Forestry.

[17] Elith J., Leathwick J.R. (2009). Species distribution models: Ecological explanation and prediction across space and time. Annu. Rev. Ecol. Evol. Syst..

[18] Dormann C.F., Schymanski S.J., Cabral J., Chuine I., Graham C., Hartig F., Kearney M., Morin X., Römermann C., Schröder B. (2012). Correlation and process in species distribution models: Bridging a dichotomy. J. Biogeogr..

[19] Guisan A., Thuiller W. (2005). Predicting species distribution: Offering more than simple habitat models. Ecol. Lett..

[20] Cutler D.R., Edwards T.C., Beard K.H., Cutler A., Hess K.T., Gibson J., Lawler J.J. (2007). Random forests for classification in ecology. Ecology.

[21] Sutherst R.W., Maywald G.F. (1985). A computerised system for matching climates in ecology. Agric. Ecosyst. Environ..

[22] Kearney M., Porter W. (2009). Mechanistic niche modelling: Combining physiological and spatial data to predict species’ ranges. Ecol. Lett..

[23] Pertierra L.R., Bartlett J.C., Duffy G.A., Vega G.C., Hughes K.A., Hayward S.A.L., Convey P., Olalla-Tarraga M.A., Aragón P. (2020). Combining correlative and mechanistic niche models with human activity data to elucidate the invasive potential of a sub-Antarctic insect. J. Biogeogr..

[24] Jung J.M., Byeon D.H., Lee D.H., Nam Y., Jung S., Lee W.H. (2023). Spatial analysis of the occurrence of the western conifer seed bug *Leptoglossus occidentalis* (Heteroptera: Coreidae) in Europe based on multiple environmental variables. Ecol. Evol..

[25] Kriticos D.J., Maywald G.F., Yonow T., Zurcher E.J., Herrmann N.I., Sutherst R.W. (2015). CLIMEX Version 4: Exploring the Effects of Climate on Plants, Animals and Diseases.

[26] Kramer-Schadt S., Niedballa J., Pilgrim J.D., Schröder B., Lindenborn J., Reinfelder V., Stillfried M., Heckmann I., Scharf A., Augeri D. (2013). The importance of correcting for sampling bias in MaxEnt species distribution models. Divers. Distrib..

[27] Jia Z.Y., Chen C., Ma Y.X., Li S.Y., Fan B.Q., Wang Y., Hao D.J. (2020). Effects of temperature on growth and development of *Pagiophloeus tsushimanus* Morimoto. J. Nanjing For. Univ. (Nat. Sci. Ed.).

[28] Jung J.M., Lee W.H., Jung S. (2016). Insect distribution in response to climate change based on a model: Review of function and use of CLIMEX. Entomol. Res..

[29] GBIF Occurrence Download.

[30] Google Google Maps. https://www.google.com/maps.

[31] GBIF Occurrence Download.

[32] Zhang L., Jing Z., Li Z., Liu Y., Fang S. (2019). Predictive modeling of suitable habitats for *Cinnamomum camphora* (L.) Presl using Maxent model under climate change in China. Int. J. Environ. Res. Public Health.

[33] Meng J., Li M., Guo J., Zhao D., Tao J. (2021). Predicting suitable environments and potential occurrences for *Cinnamomum camphora* (Linn.) Presl. Forests.

[34] Boria N.E., Olson L.E., Goodman S.M., Anderson R.P. (2014). Spatial filtering to reduce sampling bias can improve the performance of ecological niche models. Ecol. Model..

[35] Brown J.L., Bennett J.R., French C.M. (2017). SDMtoolbox 2.0: The next generation Python-based GIS toolkit for landscape genetic, biogeographic and species distribution model analyses. PeerJ.

[36] Fick S.E., Hijmans R.J. (2017). WorldClim 2: New 1-km spatial resolution climate surfaces for global land areas. Int. J. Climatol..

[37] Kennedy C.M., Oakleaf J.R., Theobald D.M., Baruch-Mordo S., Kiesecker J. (2019). Managing the middle: A shift in conservation priorities based on the global human modification gradient. Glob. Change Biol..

[38] Venter O., Sanderson E.W., Magrach A., Allan J.R., Beher J., Jones K.R., Possingham H.P., Laurance W.F., Wood P., Fekete B.M. (2016). Sixteen years of change in the global terrestrial human footprint and implications for biodiversity conservation. Nat. Commun..

[39] Tatem A.J. (2017). WorldPop, open data for spatial demography. Sci. Data.

[40] O’Neill B.C., Tebaldi C., van Vuuren D.P., Eyring V., Friedlingstein P., Hurtt G., Knutti R., Kriegler E., Lamarque J.F., Lowe J. (2016). The Scenario Model Intercomparison Project (ScenarioMIP) for CMIP6. Geosci. Model Dev..

[41] Jones B., O’Neill B.C. (2016). Spatially explicit global population scenarios consistent with the Shared Socioeconomic Pathways. Environ. Res. Lett..

[42] Thuiller W., Lafourcade B., Engler R., Araújo M.B. (2009). BIOMOD—A platform for ensemble forecasting of species distributions. Ecography.

[43] R Core Team (2025). R: A Language and Environment for Statistical Computing.

[44] Barbet-Massin M., Jiguet F., Albert C.H., Thuiller W. (2012). Selecting pseudo-absences for species distribution models: How, where and how many?. Methods Ecol. Evol..

[45] Fielding A.H., Bell J.F. (1997). A review of methods for the assessment of prediction errors in conservation presence/absence models. Environ. Conserv..

[46] Swets J.A. (1988). Measuring the accuracy of diagnostic systems. Science.

[47] Allouche O., Tsoar A., Kadmon R. (2006). Assessing the accuracy of species distribution models: Prevalence, kappa and the true skill statistic (TSS). J. Appl. Ecol..

[48] Jenks G.F. (1967). The data model concept in statistical mapping. Int. Yearb. Cartogr..

[49] Liu C., Berry P.M., Dawson T.P., Pearson R.G. (2005). Selecting thresholds of occurrence in the prediction of species distributions. Ecography.

[50] Kriticos D.J., Webber B.L., Leriche A., Ota N., Macadam I., Bathols J., Scott J.K. (2012). CliMond: Global high-resolution historical and future scenario climate surfaces for bioclimatic modelling. Methods Ecol. Evol..

[51] New M., Lister D., Hulme M., Makin I. (2002). A high-resolution data set of surface climate over global land areas. Clim. Res..

[52] Pachauri R.K., Reisinger A., IPCC, Core Writing Team (2007). Climate Change 2007: Synthesis Report. Contribution of Working Groups I, II and III to the Fourth Assessment Report of the Intergovernmental Panel on Climate Change.

[53] Hawkins E., Sutton R. (2009). The potential to narrow uncertainty in regional climate predictions. Bull. Am. Meteorol. Soc..

[54] Choat B., Jansen S., Brodribb T.J., Cochard H., Delzon S., Bhaskar R., Bucci S.J., Field T.S., Gleason S.M., Hacke U.G. (2012). Global convergence in the vulnerability of forests to drought. Nature.

[55] Branco M., Nunes P., Roques A., Fernandes M.R., Orazio C., Jactel H. (2019). Urban trees facilitate the establishment of non-native forest insects. NeoBiota.

[56] Wisz M.S., Pottier J., Kissling W.D., Pellissier L., Lenoir J., Damgaard C.F., Dormann C.F., Forchhammer M.C., Grytnes J.A., Guisan A. (2013). The role of biotic interactions in shaping distributions and realised assemblages of species: Implications for species distribution modelling. Biol. Rev..

[57] Pearse I.S., Altermatt F. (2013). Predicting novel trophic interactions in a non-native world. Ecol. Lett..

[58] De Frenne P., Zellweger F., Rodríguez-Sánchez F., Scheffers B.R., Hylander K., Luoto M., Vellend M., Verheyen K., Lenoir J. (2019). Global buffering of temperatures under forest canopies. Nat. Ecol. Evol..

[59] Titeux N., Henle K., Mihoub J.B., Regos A., Geijzendorffer I.R., Cramer W., Verburg P.H., Brotons L. (2016). Biodiversity scenarios neglect future land-use changes. Glob. Change Biol..

[60] Haack R.A., Britton K.O., Brockerhoff E.G., Cavey J.F., Garrett L.J., Kimberley M., Lowenstein F., Nuding A., Olson L.J., Turner J. (2014). Effectiveness of the international phytosanitary standard ISPM No. 15 on reducing wood borer infestation rates in wood packaging material entering the United States. PLoS ONE.

[61] Rassati D., Faccoli M., Petrucco Toffolo E., Battisti A., Marini L. (2015). Improving the early detection of alien wood-boring beetles in ports and surrounding forests. J. Appl. Ecol..

[62] Jactel H., Brockerhoff E.G. (2007). Tree diversity reduces herbivory by forest insects. Ecol. Lett..

